# Community Assembly of Adult Odonates in Tropical Streams: An Ecophysiological Hypothesis

**DOI:** 10.1371/journal.pone.0123023

**Published:** 2015-04-23

**Authors:** Paulo De Marco Júnior, Joana Darc Batista, Helena Soares Ramos Cabette

**Affiliations:** 1 Laboratorio de Teoria, Metacomunidades e Ecologia de Paisagens, Departamento de Ecologia, ICB, Universidade Federal de Goiás, Goiânia, GO, Brasil; 2 Laboratório de Entomologia, Universidade do Estado de Mato Grosso (UNEMAT), Nova Xavantina, MT, Brasil; 3 Departamento de Biologia, Universidade do Estado de Mato Grosso (UNEMAT), Nova Xavantina, MT, Brasil; Universidade de São Paulo, Faculdade de Filosofia Ciências e Letras de Ribeirão Preto, BRAZIL

## Abstract

Community assembly theory is founded on the premise that the relative importance of local environmental processes and dispersal shapes the compositional structure of metacommunities. The species sorting model predicts that assemblages are dominated by the environmental filtering of species that are readily able to disperse to suitable sites. We propose an ecophysiological hypothesis (EH) for the mechanism underlying the organization of species-sorting odonate metacommunities based on the interplay of thermoregulation, body size and the degree of sunlight availability in small-to-medium tropical streams. Due to thermoregulatory restrictions, the EH predicts (i) that larger species are disfavored in small streams and (ii) that streams exhibit a nested compositional pattern characterized by species’ size distribution. To test the EH, we evaluate the longitudinal distribution of adult Odonata at 19 sites in 1^st^- to 6^th^-order streams in the Tropical Cerrado of Brazil. With increasing channel width, the total abundance and species richness of Anisoptera increased, while the abundance of Zygoptera decreased. The first axis of an ordination analysis of the species abundance data was directly related to channel width. Mean and maximum thorax size are positively correlated to channel width, but no relationship was found for the minimum thorax size, suggesting that there is no lower size constraint on the occurrence of these species. Additionally, a nested compositional pattern related to body size was observed. Our results support the EH and its use as an ecological assembly rule based on abiotic factors. Forest cover functions as a filter to determine which species successfully colonize a given site within a metacommunity. As a consequence, the EH also indicates higher treats for small-bodied zygopterans in relation to the loss of riparian forests in tropical streams.

## Introduction

One key aspect of current community assembly theory is the relative importance of local environmental factors and dispersal processes shaping compositional patterns within metacommunities. Four general models describe interesting combinations of these factors and are frequently used to interpret observed communities [1]: neutral model, patch dynamics, mass-effect and species sorting. Only the neutral theory proposes that the habitat requirements of different species do not play any important role, with dispersal constraints posited as the dominant factor structuring community assembly [2]. The patch dynamic model predicts the existence of some empty suitable patches due to low dispersal rates [1,3]. Conversely, the mass-effect model is applicable when dispersal rates are so high that even less-suitable habitats are occupied in a given period. Finally, the species sorting model is based on a dispersal rate that is sufficient to enable every species to reach suitable habitats and posits that niche requirements play the dominant role determining the species composition of a given assemblage. Under the last perspective, it is expected that environmental filtering can be traced through the identification of local environmental variables that determine habitat quality for a given assemblage. Moreover, it is also expected that a species will be highly adapted to the habitat conditions in which its existence is favored.

Environmental gradients are highly useful for evaluating metacommunity models, especially from a species sorting perspective [4,5]. Some human-generated gradients (*e*.*g*., those induced by land-use changes) may allow broad predictions to be made (for example, that generalist species are favored in altered habitats [6],) but many studies have also demonstrated the association of particular traits with successional [7] or other environmental gradients [8,9]. For instance, river basins are expected to present strong and predictable longitudinal variation related to stream/river size [10]. The River Continuum Concept (RCC, [11]) is mostly based on this generally observed pattern and remains one of the best theoretical tools for describing these changes and interpreting the longitudinal distribution of insects, at least from headwater streams to medium rivers [12]. Following the classic paper by Vannote et al. [11], we expect directional changes in rivers to result from the balance of the energy captured and imported from river margins. Channel width is the key factor that determines the amount of solar energy that enters the system and the relative importance of imported energy in river metabolism from the margins. Thus, small streams are strongly affected by riparian forests [11,13] and depend on allochthonous material to support a heterotrophic food chain [14]. As channel width increases, the importance of riparian vegetation decreases [15], while primary production increases as a function of the increase in direct solar radiation ([11],but see [16]). Cummins & Klug [14] and Vannote et al. [11] predicted a longitudinal distribution of aquatic invertebrate guilds, with shredders, which depend on CPOM (coarse particulate organic matter), being more abundant and diverse in headwaters, and collectors, which use FPOM (fine particulate organic matter), being more abundant and diverse in medium and large rivers.

The general theory of longitudinal distribution successfully explains the distribution of macroinvertebrate guilds, especially concerning detritivores and shredders [12], but does not generate critical distribution predictions for predators, which are expected to be more or less regularly distributed along a river [11] or to suffer a small decrease in abundance near headwaters [17]. Otherwise, many stream-dwelling insect predators, such as Coleoptera, Heteroptera, and Odonata, exhibit terrestrial adults. This condition introduces a different set of environmental variables not previously included in the RCC theory, which may affect their population dynamics. For instance, many odonate species present territorial adults that defend areas at or near stream margins and are influenced by the height of riparian vegetation and solar radiation [18,19]. The thermoregulatory abilities of the adults may represent important eco-physiological barriers that could explain their habitat choices [20–22] and constrain the larval distribution in streams. Here, we propose an ecophysiological hypothesis (EH) for the main mechanism determining species sorting in odonate metacommunities. This hypothesis posits that size-related thermoregulatory constraints on habitat use are the main filter for community assembly from headwaters to medium-sized tropical streams.

The evolutionary patterns of odonate thermoregulation are driven by interactions in endothermy, body size and behavior. Some medium and large-sized odonates are capable of endothermic warm-up most related to wing whirring, and can maintain body temperature by controlling haemolymph circulation [23,24]. Small odonates exhibit a higher surface area/volume ratio and are usually ectothermic and highly dependent on convection heat exchange for activity [25–27]. These species present bodies with a higher conductance, and their body temperatures vary according to the air temperature, hence being considered thermal conformers [28]. As size increases in cross-species comparisons, the total surface is more important for determining the relative efficiency of convective and irradiative heat exchange. Thus, larger ectothermic species are expected to exhibit heliothermic behaviour and to be more dependent on direct radiation to heat their thoracic muscles for flight [26]. All of these species usually remain perched for the larger portion of their activity time and are recognized as “perchers”[27]. However, endothermic species are expected to be larger and to exhibit more “flier” behaviour, remaining on the wing for the majority of their active time.

Considering these ecophysiological constraints, we expect larger ectothermic species (heliotherms) to be favored in open areas of streams and lakes allowing direct exposure to the sun. It has long been recognized that forested, shaded habitats maintain a steady temperature [29], especially in tropical areas of the world. A warm and steady temperature favors small ectothermic species (conformers). In small headwater streams, there is a strong shade effect of the riparian forest compared with medium-sized rivers, and our EH predicts dominance of small odonate species. In medium-to-larger portions of a river system (with a greater channel width), we expect an increase in the abundance of larger odonates. An important assumption of this reasoning is that larger, heliothermic species are severely limited from using small, forested streams. Additionally, we expect small, conformer species to be regularly distributed throughout the system because they may exhibit behavioral mechanisms to address an increase in sun exposure [20,21,30–33]. This assumption leads to the prediction of a nested pattern [34] in the species distribution of odonate stream assemblages: increased channel width may increase the probability of colonization for larger species, but small species would be maintained. The maximum size in these assemblages would be most likely determined by the regional species pool, and we expect the maximum observed size to show an asymptotic response curve in relation to channel width. We also expect an increase in the mean body size in relation to channel width, but the minimum observed size should remain unchanged. Finally, we recognize the stream-dwelling odonate metacommunity as an example of a species-sorting process, based first on all of the above-predicted relationships between individual species traits (mostly body size) and the thermal environment. Additionally, it is easy to assume one of the key aspects of this model [1], namely the ability of individual species to arrive at suitable sites through dispersal, which is a logical consequence of the ability of odonates to fly.

The order Odonata is represented by two suborders in the Neotropical region. Zygoptera are typically small-sized, slender species, mainly classified as thermal conformers [21,35,36]. Anisoptera show a medium-to-larger size and are mainly classified as heliothermic, but with some thermal conformers and endothermic species [27,35,37]. These broad distinctions between these groups aid in the development of specific predictions of their distribution according to the EH. Thus, we evaluate the explanatory power of the EH through analysis of the regional distribution of ectothermic Odonata in a Central Brazilian river basin. Although the EH facilitates a large number of theoretical expectations, we consider that its main predictions are (i) that larger species should be disfavored in small forested streams due to their heliothermy; and (ii) that forested streams should display a nested pattern of the species distribution of these communities according to their body sizes. From (i), it is also expected that (i_1_) an increase of the maximum size should be observed in relation to channel width, whereas no relationship should be found for the minimum observed size; and (i_2_) a decrease of Zygoptera species and an increase of Anisoptera species should be observed in relation to increasing channel width.

## Materials and Methods

### Study Area

**Fig 1:**
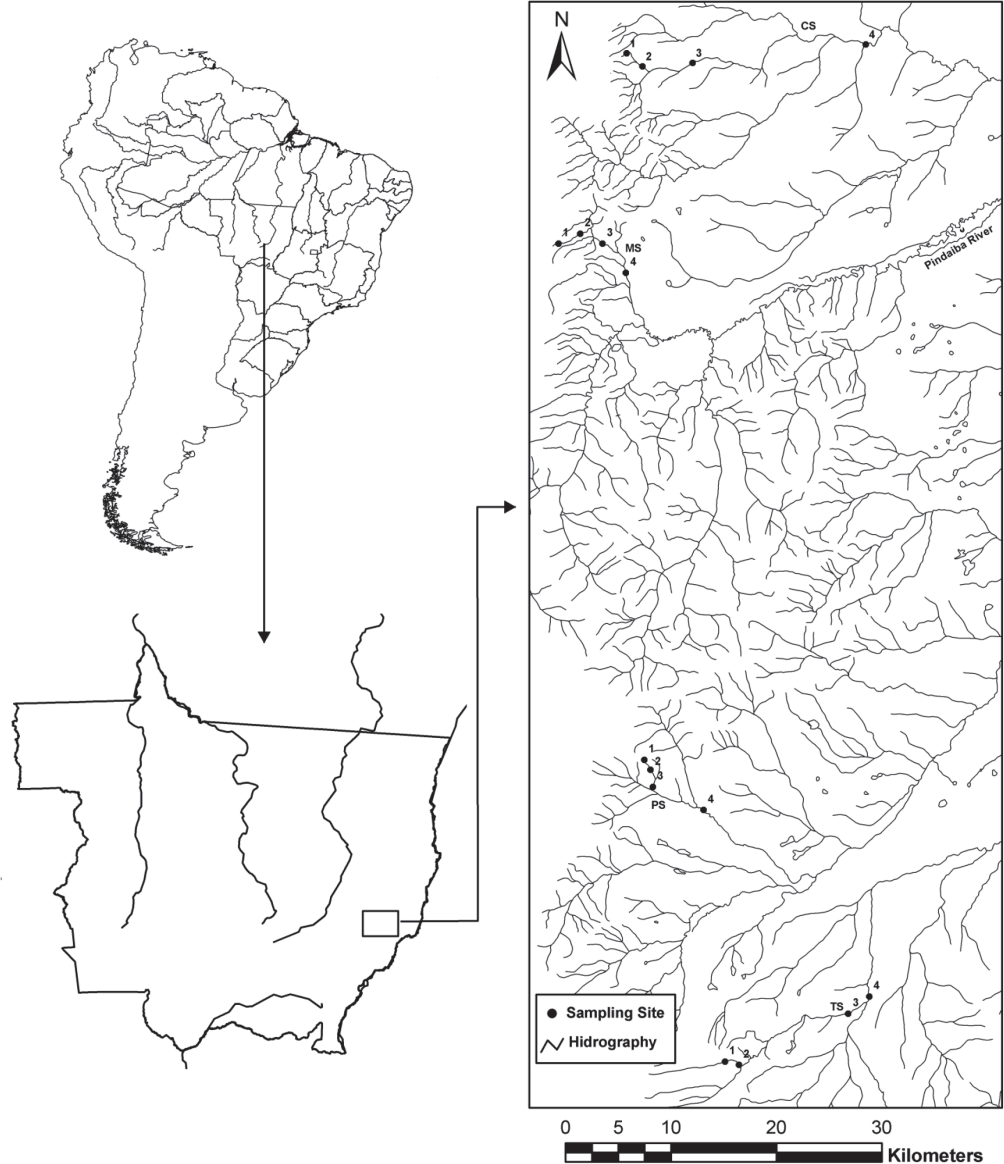
Location of the study sites in the Pindaíba River basin, Mato Grosso, Brazil. CS, Cachoeirinha Stream; MS, Mata Stream; PS, Papagaio Stream; and TS, Taquaral Stream. The numbers represent stream orders.

The Pindaíba river basin is part of the Mortes river basin and is located in the southwest of Mato Grosso State, Central Brazil. The basin exhibits a surface area 10.323 km^2^ and includes the municipalities of Barra do Garças, Araguaiana, Cocalinho and Nova Xavantina (Fig 1). The system drains predominantly from south to north, with the majority of the headwaters occurring in areas with an altitude of 600 m and the medium river systems being found in areas with a 330 m mean altitude. The main economic activities in this area are cattle ranching and soybean cultivation.

This study was conducted at 19 sites in the Pindaíba river basin in the Mata (1^st^ to 4^th^ orders), Cachoeirinha (1^st^ to 3^rd^ order), Zacarias (4^th^ order), Papagaio (1^st^ to 4^th^ order), Taquaral (1^st^ to 3^rd^ order) and Ìnsula (4^th^ order) streams and the Corrente river (5^th^ order) and Pindaíba river (5^th^ and 6^th^ order). The stream order was based on the classification of Strahler [38]. In our analysis, we use channel width, which varied at least two orders of magnitude among the sampled sites (1.0 to 5.5 m), as the best descriptor of the river system. The choice of these sampling sites followed two basic criteria related to the independent variables assessed in our study. First, we aimed to include an acceptable variability in channel width among the sampling units. Second, we ensured that all of the sites have well-preserved riparian vegetation, which is one of the basic assumptions of our hypothesis. We consider a patch of riparian area as “well-preserved” if there is observed a preserved understory (no indication of cattle invasion), presence of large trees and continuous forest presence in a strip higher than 500m at the side of the stream. Finally, all sites of the same order were in different branches of the river system (Fig 1) to ensure the independence of samples in the statistical analysis.

### Biological samples

Quantitative sampling of odonates was performed using the fixed-area scan method [39–41]. All adults observed in a 100 m stream segment were recorded in short scans during one minute. This segment was divided into 20 segments of 5 m each, and the one-day observations were repeated in the rainy (January/February) and dry seasons (July/August). Sampling activity was restricted to days with clear skies and temperatures above 19°C and was performed between 10 AM and 2 PM to avoid bias due to species-specific activity patterns [37]. This sampling design is less affected by problems such as double-counting individuals partially because the short time-intervals counts in small segments, but also because the low population density for all species in these forested streams. The species were identified according to specialized keys [42–45] and compared with the collection of the Theoretical Ecology and Synthesis Laboratory of the Federal University of Goiás. The last author, HSRC, has a permanent license to collect aquatic insects (14457–1) provided by IBAMA/Sisbio, in accordance with federal law and the regulations of the Brazilian Environmental Ministry. The sampling sites were private, and permission from the owner or manager was obtained prior to sampling. None of the sampled species were protected by Brazilian law or red-listed.

### Data Analysis

We consider each stream as a replicate for the statistical analysis. River order is generally used as a statistical variable against which longitudinal patterns in rivers are tested [12,46]; however, this is not the best functional measure of the mechanism underlying our hypothesis. As the EH is a consequence of the importance of the input of light radiation to the system, channel width appears to be a more appropriate variable for this study. Moreover, the use of stream order may produce misleading results because some second-order streams exhibit widths that allow an increased incidence of sunlight, even in areas where the riparian forest is preserved.

Observed species richness has long been recognized to be intrinsically biased [47–49]. Thus, we employ a jackknife estimate of species richness using the segments as replicates in each stream, according to Cowell & Coddington [50]. The relationship between species richness and channel width was tested using standard linear regression techniques [51], and the possible dependency between the proportion of Zygoptera species and channel width was tested using logistic regression [52].

Our hypothesis concerning the arrangement of the assemblage composition in relation to body size requires an appropriate measure of size. Considering odonate physiology, we use the thorax length as a reliable measure of “size”. The size of thoracic muscles is a key feature determining the thermoregulatory capacity of these species and is expected to be directly correlated with both the amount of heat needed to initiate flight and the amount of heat that can be produced during flight [26]. We measured thorax length using a stereomicroscope with calipers for at least 5 individuals of each species selected from the collected individuals in the area and the odonate collection of the Federal University of Goiás.

We evaluate the basic predictions of the EH using two different approaches. First, we apply a detrended correspondence analysis (DCA) [53] to obtain an ordination of the assemblages and test the relationship of the first axis with the mean body sizes for each site via standard linear regression. Second, we estimate mean, minimum and maximum body sizes at each site and test for the existence of dependency on channel width according to the specific predictions of the EH. A non-linear relationship between the maximum observed size and channel width was estimated using the following model:

$$W_{\text{max}}=W_{\infty }(1-e^{aC})$$ (Eq 1)

where *W*_max_ is the maximum observed size; *W*_*∞*_ is the maximum size in the regional species pool; *a* is a parameter that measures the effect of the channel width; and *C* is the channel width.

Eq 1 represents only one possible model of a non-linear asymptotic relationship that could be expected under our hypothesis. We selected this equation because it is the simplest model for this relationship, with the smallest number of parameters. We fit this model using nonlinear estimation procedures with least square loss function using a quasi-Newtonian optimization procedure with Statistica 8.0 (Statsoft, Inc.; Chicago, USA).

To test the existence of a nested pattern of community structure attributable to the EH, we evaluated the NODF index (Nestedness metric based on Overlap and Decreasing Fill) on the whole community matrix ordered from small to large species in the columns and from small to large streams in the rows. This measure is independent of other nuisance factors that affect these analyses, such as the shape and size of the matrix [54]. We estimate statistical significance for NODF by comparing the observed values against a CE null model [55] of the community matrix. Under this algorithm, the probability of the occurrence of a species at a given site is the average of the probability of choosing this species at random (a function of its frequency) and the probability of choosing this site at random (a function of its species richness).

## Results

**Fig 2:**
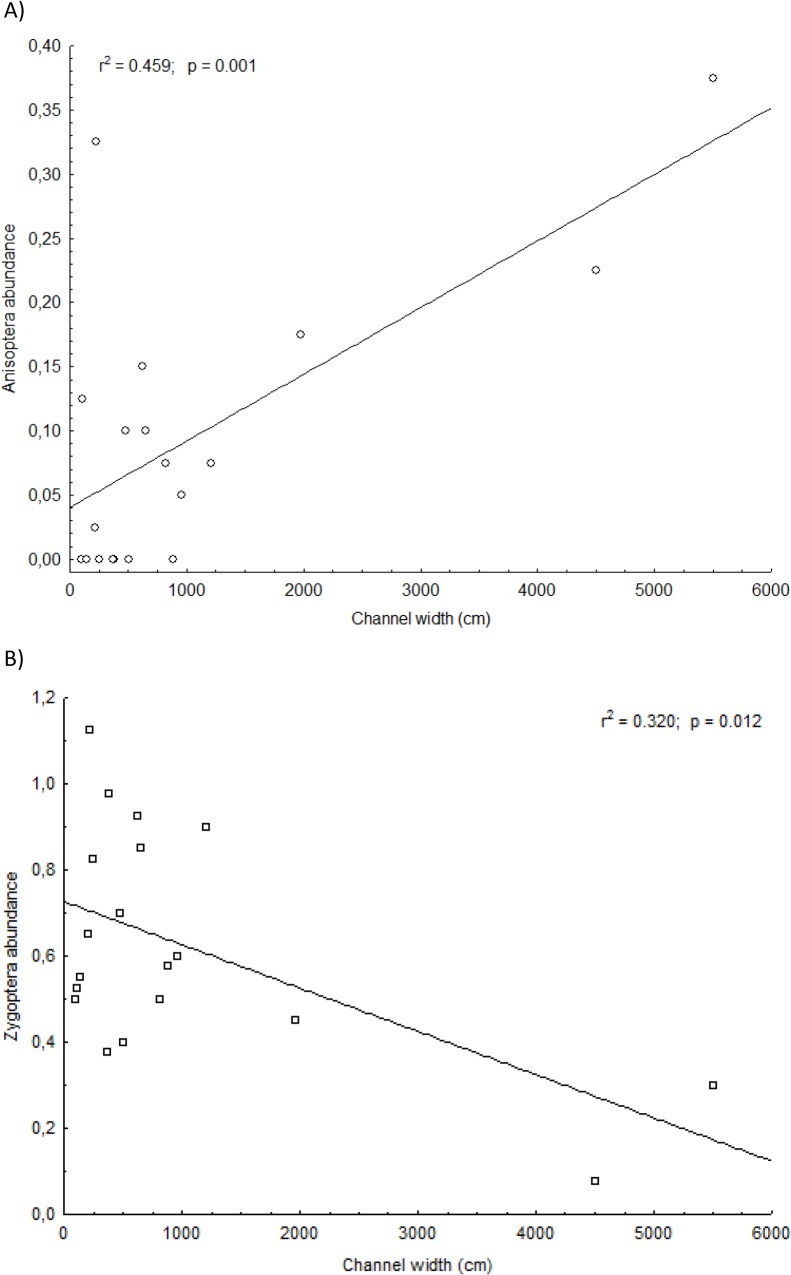
Linear regression of Zygoptera (A) and Anisoptera (B) abundance against channel width in the Pindaíba river basin (Mato Grosso, Brazil) samples.

The taxonomic composition of the sampled sites showed a higher representation of Zygoptera (seven families, 14 genera and 28 species) than Anisoptera (only one family (Libellulidae), with 13 genera and 17 species). The total abundance of Anisoptera increased (R^2^ = 0.318, p = 0.012 Fig 2A) and the abundance of Zygoptera decreased (R^2^ = 0.339, p = 0.009, Fig 2B) in relation to channel width, as predicted by the EH.

**Fig 3:**
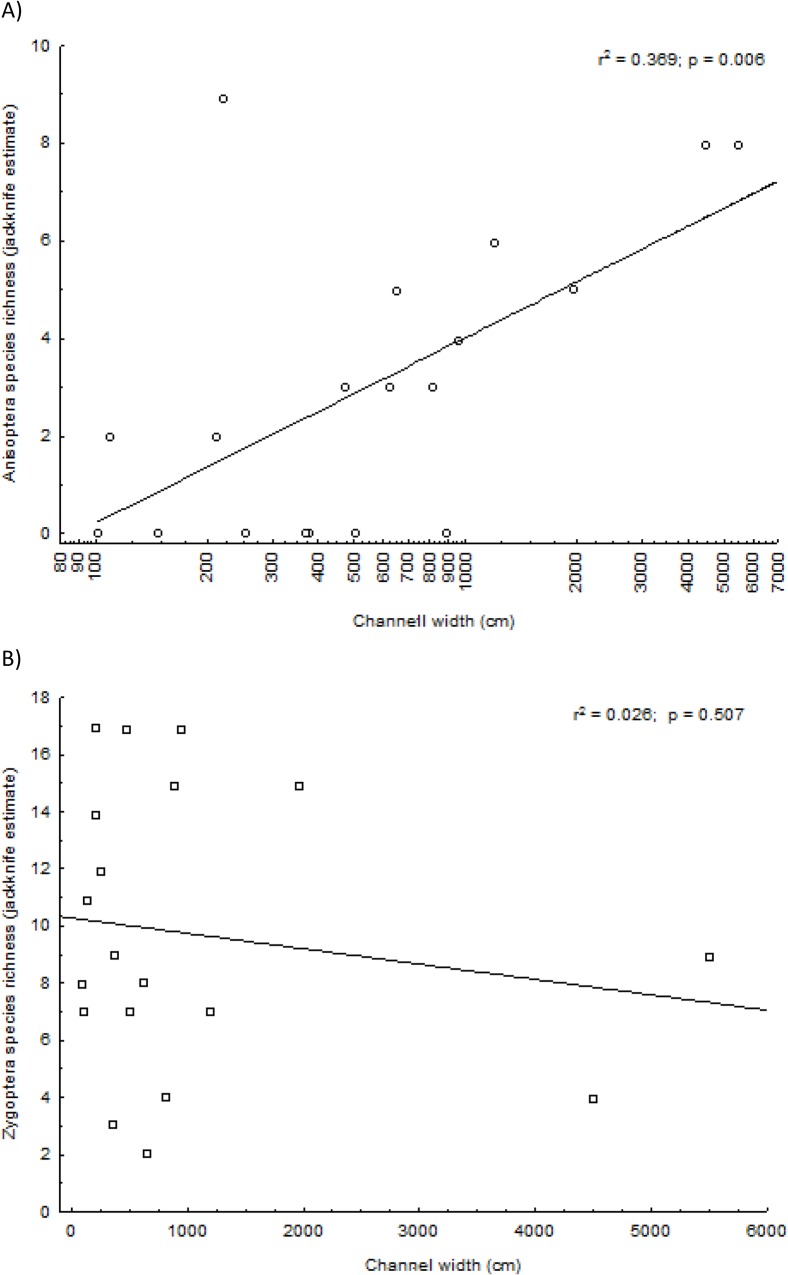
Linear regression of Zygoptera (A) against log-transformed channel width and Anisoptera (B) abundance against raw channel width data in the Pindaíba river basin (Mato Grosso, Brazil) samples.

The residual analysis of the linear regression between Anisoptera species richness and channel width showed a distribution pattern consistent with a non-linear response. Thus, we demonstrate this relationship with the log-transformed channel width (Fig 3A). Anisoptera species richness was clearly affected by channel width (Fig 3A, R^2^ = 0.369 p = 0.006), but there was no effect for Zygoptera species richness (Fig 3B, R^2^ = 0.026, p = 0.507). A non-linear relationship with Anisoptera should arise from constraints imposed by the size of the species pool available to colonize these systems.

**Fig 4:**
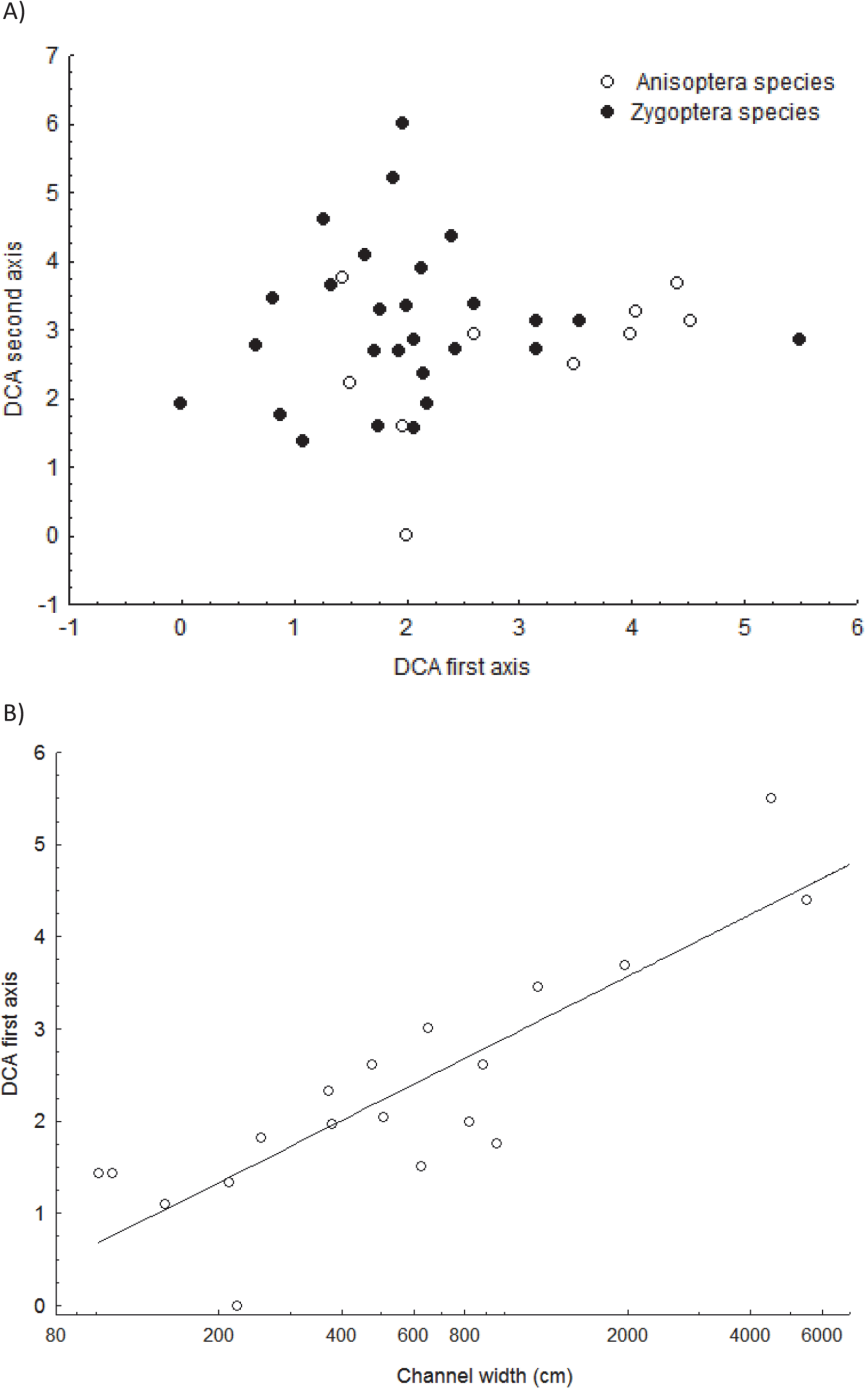
Detrended correspondence analysis for the odonate assemblage in the Pindaiba stream system (A) and the regression of the first DCA axis with channel width (Y = 1.566+0.224*log10(x); R^2^ = 0.853; p<0.001).

Detrended correspondence analysis showed that Anisoptera species presented higher scores on the first axis in relation to Zygoptera species (*t*-test of variance = 2.193; df = 23; p = 0.038, Fig 4A). Channel width, on which the first DCA axis loaded heavily, is a determinant of species composition. This result provides evidence of a decrease of Zygoptera species abundance in larger streams (DCA1 = 1.566+0.224*log_10_(channel width); R^2^ = 0.853; p<0.001; Fig 4B).

**Fig 5:**
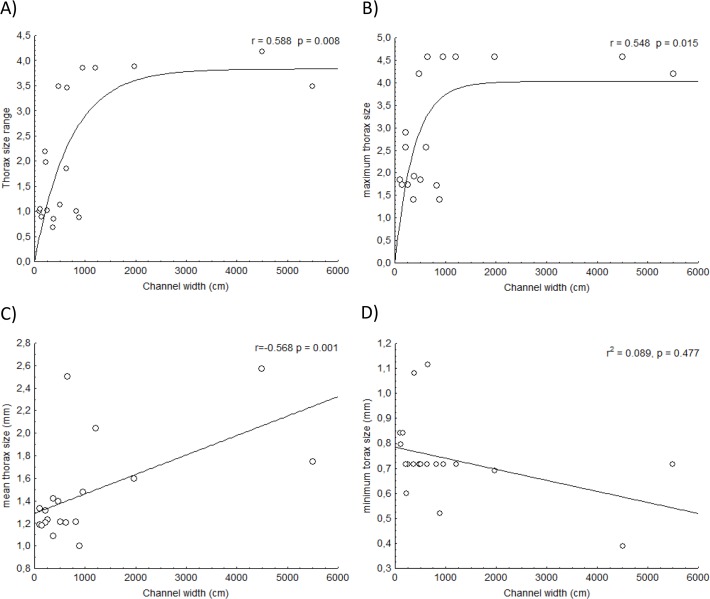
Relationship of different community body-size measures with channel width. For the range (maximum—minimum) of thorax size (A) and maximum values (B) in each assemblage, a sigmoid curve was fitted. For the minimum (C) and mean thorax size (D), a linear relationship was fitted to the data.

The general relationship between body size metrics and channel width showed a clear assembly rule for the species composition in these systems. First, there was a strong relationship between the range (Fig 5A), maximum (Fig 5B) and mean thorax size (Fig 5C) with channel width. However, there was no correlation of minimum thorax size (Fig 5D) on channel width, suggesting that there is no lower size limit that determines species presence. These results suggest that larger-sized species are added to the community as channel width increases. The existence of a constraint imposed by the number of larger species present in the species pool that may colonize a given site is the primary cause of the non-linear relationships observed in Fig 5A and 5B. This general result supports the EH and suggests a special constraint for larger species to colonize small forest-covered streams.

The NODF value was estimated to be 17.13, which was higher than expected by chance (CE null model prediction = 13.84; p = 0.020). Considering only the Zygoptera, the value increased to 20.69 and was also higher than expected by chance (CE null model prediction = 14.99; p = 0.010).

## Discussion

Our results support an eco-physiological hypothesis for the odonate species distribution in this stream system. Narrow streams are more affected by riparian vegetation, which reduces the light input but also generates a more stable thermal environment. These characteristics favored small species that were less dependent on direct sunlight exposure for their thermoregulation [26,37] but limited the colonization of larger, heliothermic species. The taxonomic distinction between small streams (dominated by Zygoptera) and larger rivers (by Anisoptera) is an incidental consequence of differences in body size and associated thermal properties between these two groups.

Different ecological models attempt to explain patterns of community structure in local communities based on synthetic ecological parameters such as species diversity, species richness or functional diversity [2,56–59]. Moreover, only a limited number of theories suggest useful hypotheses about the species composition of local assemblages, which is a far more complex issue [1,5,60,61]. The best approach for such an enterprise is most likely the “assembly rules” approach pioneered by Diamond [62] and subsequently developed into a multitude of possible solutions [60,63–65]. Here, we are especially interested in a subset of those rules, specifically the niche-based assembly rules [66], which are expected to determine the composition of species-sorting metacommunities [1]. We safely assume that these rules can be separated into at least two conceptual sets. Interactive assembly rules, the first set, presume that the initial composition of the community may determine which species are capable of colonizing this system [67]. Our EH belongs to the second set, and presumes that abiotic factors—in this case temperature, mediated by forest cover—function as filters that determine which species may be successful in a given community (following a gleasonian paradigm [68]), or at least as important factors determining the outcomes of interactive processes, such as competition [69]. We expect that some species-specific traits (life-history, physiological, morphological or behavioral traits) may play an important role in the interaction of individual species with these filters. Recently, Kadoya et al. [70] followed a similar theoretical approach and hypothesized that long-lived odonate species should be more sensitive to habitat fragmentation. Thus, trait-based explanations for the response of individual species to environments, such as this EH, are our best choice to produce useful predictions of the community composition, at least in systems with a limited influence of interactive factors.

Nested patterns of species composition are one important component of beta diversity [71]. Nevertheless, it is not easy to identify the mechanisms responsible for such patterns in many cases. Here, we could associate the nested patterns of the community composition of odonates in streams with species’ ecophysiological responses to their environment, mainly to sunlight. Corbet & May (2008) showed that small perchers “are relatively unlikely to overheat because they are subject to high rates of convective heat loss, but may sometimes have difficulty attaining a high Tb (body temperature)”. This may be the basic mechanism underlying nestedness in these communities, as small perchers (usually Zygoptera) were unaffected by the higher energy load of larger streams, and larger perchers were added in those sites. This generated a predictable size structure of the community and its compositional changes. The basic aspects of this theory appear sufficiently robust to generate strong generalizations, mainly because it results from a physical law (how irradiative heat may be related to body size) and an ecological fact (the differences in body size among species).

Thermoregulatory abilities are not a restricted function of body size. Other important factors, such as behavioral characteristics [20,72] and color [32], directly affect the response of odonate species to their thermal environments. For instance, Damm et al. [73] showed an association between dark-colored species and forested environments during the evolution of the *Trithemis* genus in Africa. The relative importance of behavioral or color adaptations in relation to body size effects expected under the EH is difficult to predict, but there is no reason for those different mechanisms not to work together. In fact, considering our theory, the hypothesis that dark *Trithemis* species associated with forested habitats are also smaller is particularly interesting and warrants further investigation.

Many observed patterns of species compositions or responses to environmental variation in tropical odonate communities could be interpreted as evidence for the EH and may provide additional support for our findings. For instance, the increase in the larger species *Hetaerina rosea* recorded in altered habitats and the increase of the small shade-seeking *Heteragrion aurantiagrum* in Atlantic forest streams [74] could be explained by this hypothesis. The dominance of small Zygoptera in forested Amazonian headwater streams together with the increased presence of Anisopteran species in third-order streams [18] can be explained similarly. Moreover, a lower abundance of *Trithemis* species (which exhibit a comparatively larger body size) at sites with high or moderate vegetation cover in ponds [75] further support our hypothesis, and forested streams in Kenya are also dominated by Zygoptera species [76], which is predicted by our hypothesis. Thus, the EH generalizes well, at least in tropical systems. Moreover, the EH may be tested in temperate streams, and it may contribute as a partial explanation related to life-history attributes, in conjunction with other trait-based explanations [70,77].

The RCC theory predicts a higher diversity in intermediate portions of river systems [11]. However, we observed a general increase of species richness, at least for the Anisoptera. Nevertheless, the original prediction was for the entire fauna and not only for a single group. This general theory about river function was initially proposed based on rivers in temperate climates, in which important variables such as temperature, riparian forest diversity, rainfall and evolutionary histories may have very different effects [78]. Tropical streams present stable temperatures during the day and throughout the year [79]; a decrease of temperature variation associated with increasing stream size is one of the important bases of the RCC theory. Although RCC theory remains an important general model for river function [80], there are some important criticisms about its basic assumptions [81] and the validity of some of its predictions [79,82]. Our results suggest that a possible new way in which to explore this hypothesis is to discuss species whose entire life history takes place in aquatic systems separately from those that have terrestrial adults (such as the Odonata, Coleoptera and Heteroptera). It has been recently demonstrated that such a split life history in a group may increase local extinction rates [83,84] and may entail different environmental forces that may produce alternative stable strategies for microhabitat selection in the group.

Another important consequence of the EH is that we expect the substitution of smaller species with larger species, e.g., zygopterans with anisopterans, when small stream riparian vegetation is altered or converted to other activities. This conversion is intense in large areas of Neotropical regions, mostly due to economic pressure [85–88], which suggests that thermal abilities, especially the capacity for irradiative heat exchange [24,26,89], should be considered a surrogate of species persistence in human-impacted areas, at least in the tropics. However, the dependency on convection heat exchange in small odonates should be considered a possible surrogate for predicting the threat to extinction in areas under intense land use conversion, such as some areas of the Amazon.

## References

[1] Leibold MA, Holyoak M, Mouquet N, Amarasekare P, Chase JM, Hoopes MF, et al The metacommunity concept: a framework for multi-scale community ecology. Ecol Letters 2004; 7: 601–613.

[2] Hubbell SP. The unified neutral theory of Biodiversity and Biogeography Princeton and Oxford: Princeton University Press 375 p., 2001.

[3] Winemiller KO, Flecker AS, Hoeinghaus DJ. Patch dynamics and environmental heterogeneity in lotic ecosystems. J N Amer Benth Soc 2010; 29: 84–99.

[4] Henriques-Silva R, Lindo Z, Peres-Neto PR. A community of metacommunities: exploring patterns in species distributions across large geographical areas. Ecology 2013;94: 627–639. 23687889 10.1890/12-0683.1

[5] Brown BL, Swan CM, Auerbach DA, Grant EHC, Hitt NP, Maloney KO, et al Metacommunity theory as a multispecies, multiscale framework for studying the influence of river network structure on riverine communities and ecosystems. Journal of the North American Benthological Society 2011,30: 310–327.

[6] Layer K, Hildrew AG, Woodward G. Grazing and detritivory in 20 stream food webs across a broad pH gradient. Oecologia 2013,171: 459–471. 10.1007/s00442-012-2421-x 22996363 PMC3548098

[7] Ruhi A, Boix D, Gascon S, Sala J, Quintana XD. Nestedness and successional trajectories of macroinvertebrate assemblages in man-made wetlands. Oecologia 2013,171: 545–556. 10.1007/s00442-012-2440-7 22965268

[8] Ackerly DD, Cornwell WK. A trait-based approach to community assembly: partitioning of species trait values into within- and among-community components. Ecol Letters 2007,10: 135–145. 17257101 10.1111/j.1461-0248.2006.01006.x

[9] Holdaway RJ, Sparrow AD. Assembly rules operating along a primary riverbed-grassland successional sequence. J Ecology 2006, 94: 1092–1102.

[10] Yang JA, Dilts TE, Condon LA, Turner PL, Weisberg PJ. Longitudinal- and transverse-scale environmental influences on riparian vegetation across multiple levels of ecological organization. Landsc Ecol 2011,26: 381–395.

[11] Vannote RL, Minshall GW, Cummins KW, Sedell JR, Cushing CE. The river continuum concept. Can J Fish Aquat Sci 1980,37: 130–137.

[12] Heino J, Parviainen J, Paavola R, Jehle M, Louhi P, Muotka T. Characterizing macroinvertebrate assemblage structure in relation to stream size and tributary position. Hydrobiologia 2005,539: 121–130.

[13] Fisher SG, Likens GE. Energy flow in Bear Brook New Hampshire: An integrative approach to stream ecosystem metabolism. Ecol Monogr 1973,43: 421–439.

[14] Cummins KW, Klug MJ. Feeding ecology of stream invertebrates. Annu Rev Ecol Syst 1979,10: 147–172.

[15] Conner ME, Naiman RJ. Particulate allochthonous inputs: Relationship with stream size in an undisturbed watershed. Can J Fish Aquat Sci 1984,41: 1473–1484.

[16] Franken RJM, Waluto B, Peeters ETHM, Gardeniers JJP, Beijer JAJ, Scheffer M. Growth of shredders on leaf litter biofilms: the effect of light intensity. Freshw Biol 2005,50: 459–466.

[17] Grubaugh JW, Wallace JB, Houston ES. Longitudinal changes of macroinvertebrate communities along an Appalachian stream continuum. Can J Fish Aquat Sci 1996,53: 896–909.

[18] Juen L, De Marco P Jr. Odonate beta diversity in terra-firme forest streams in Central Amazonia: On the relative effects of neutral and niche drivers at small geographical extents. Ins Conserv Diver 2011,4: 265–274.

[19] Rith-Najariam JC. The influence of forest vegetation variables on the distribution and diversity of dragonflies in a northern Minnesota forest landscape: a preliminary study (Anisoptera). Odonatologica 1998,27: 335–351.

[20] De Marco P Jr, Latini AO, Resende DC. Thermoregulatory constraints on behavior: patterns in a Neotropical dragonfly assemblage. Neotrop Entomol 2005,34: 155–162.

[21] Mckay T, Herman T. Thermoregulation in three species of damselflies, with notes on temporal distribution and microhabitat use (Zygoptera: Lestidae). Odonatologica 2008,37: 29–39.

[22] Watanabe M. Thermoregulation and habitat preference in two wing color forms of *Mnais* damselflies (Odonata, Calopterygidae). Zool Sci 1991,8: 983–989.

[23] Sformo T, Doak P. Thermal ecology of Interior Alaska Dragonflies (Odonata: Anisoptera). Func ecol 2006,20: 114–123.

[24] May ML. Energy metabolism of dragonflies (Odonata: Anisoptera) at rest and during endothermic warm-up. J Exp Biol 1979,83: 79–94.

[25] May ML. Thermoregulation in adaptation to temperature in dragonflies (Odonata: Anisoptera). Ecol Monogr 1976,46: 1–32.

[26] May ML. Thermal adaptations of dragonflies, revisited. Adv Odonat 1991,5: 71–88.

[27] Corbet PS, May ML. Fliers and perchers among Odonata: dichotomy or multidimensional continuum? A provisional reappraisal. Int J Odonatol 2008,11: 155–171.

[28] Heinrich B, Casey TM. Heat transfer in dragonflies: 'fliers' and 'perchers'. J Exp Biol 1978,74: 17–36.

[29] Matlack GR. Microenvironment variation within and among forest edges sites in the eastern United States. Biological Conservation 1993,66: 185–194.

[30] Ishizawa N. Thermoregulation of dragonflies of *Sympetrum* species in the lowlands in midsummer. Gekkan Mushi 1994,281: 13–17.

[31] May ML. Thermoregulation and reprodutive activity in tropical dragonflies of the genus *Micrathyria* . Ecology 1977,58: 787–798.

[32] May ML. Insect thermoregulation. Annu Rev Ent 1979,24: 313–349.

[33] Polcyn DM. Thermoregulation during summer activity in Mojave Desert dragonflies (Odonata: Anisoptera). Func ecol 1994,8: 441–449.

[34] Craig CN, Reece BA, McIntyre NE. Nestedness in Playa Odonates As A Function of Area and Surrounding Land-Use. Wetlands 2008,28: 995–1003.

[35] Corbet PS. Dragonflies: behavior and ecology of Odonata Ithaca, NY: Comstock Publ. Assoc. 829 p., 1999

[36] De Marco P Jr, Vital MVC. Ecology of *Tigriagrion aurantinigrum* calvert in response to variations in environmental conditions (Zygoptera: Coenagrionidae). Odonatologica 2008,37: 1–11. 18512547

[37] De Marco P Jr, Resende DC. Activity patterns and thermoregulation in a tropical dragonfly assemblage. Odonatologica 2002,31: 129–138. 12622424

[38] Strahler HN. Quantitative analysis of watershed geomorphology. Amer Geophys Union Transact 1957,33: 913–920.

[39] De Marco P Jr. The Amazonian Campina dragonfly assemblage: patterns in microhabitat use and behavior in a foraging habitat. Odonatologica 1998,27: 239–248.

[40] Silva DP, De Marco P, Resende DC. Adult odonate abundance and community assemblage measures as indicators of stream ecological integrity: A case study. Ecol Indic 2010,10: 744–752. 10.1111/j.1755-0998.2010.02834.x 21565084

[41] De Marco P Jr, Peixoto PEC. Population dynamics of *Hetaerina rosea* and its relationship to abiotic conditions (Zygoptera: Calopterygidae). Odonatologica 2004,33: 17–25.

[42] Lencioni FAA. Damselflies of Brazil, an illustrated indentification guide: II—Coenagrionidae families São Paulo, Brazil: All Print Editora, 2006.

[43] Carvalho AL, Calil ER. Chaves de identificação para as famílias de Odonata (Insecta) ocorrentes no Brasil, adultos e larvas. Pap Av Zool 41: 223–241, 2000.

[44] Borror DJ. A key to the New World genera of Libellulidae (Odonata). Ann Entomol Soc Amer 1945,38: 168–194.

[45] Lencioni FAA. Damselflies of Brazil, an illustrated indentification guide: I—The non-Coenagrionidae families São Paulo, Brazil: All Print Editora 324 p., 2005.

[46] Helson JE, Williams DD, Turner D. Larval chironomid community organization in four tropical rivers: Human impacts and longitudinal zonation. Hydrobiologia 2006,559: 413–431.

[47] Hellmann JJ, Fowler GW. Bias, precision, and accuracy of four measures of species richness. Ecol Applic 1999,9: 824–834.

[48] Walther BA, Moore J. The concepts of bias, precision and accuracy, and their use in testing the performance of species richness estimators, with a literature review of estimator performance. Ecography 2005,28: 815–829.

[49] Heltshe JF, Forrester NE. Estimating species richness using the jackknife procedure. Biometrics 1983,39: 1–11. 6871338

[50] Colwell RK, Coddington JA. Estimating terrestrial biodiversity through extrapolation. Phil Trans R Soc London 1994,345: 101–118. 7972351 10.1098/rstb.1994.0091

[51] Zar JH. Biostatistical analysis Englewood Cliffs, N.J.: Prentice-Hall 663 p., 1999.

[52] Hosmer DW, Lemeshow S. Applied logistic regression New York, Chichester, Brisbane, Toronto, Singapore: Wiley 1989

[53] Hill MO, Gauch HG. Detrended correspondence analysis, an improved ordination technique. Vegetatio 1980,42: 47–58.

[54] Almeida-Neto M, Guimaraes P, Guimaraes PR, Loyola RD, Ulrich W. A consistent metric for nestedness analysis in ecological systems: reconciling concept and measurement. Oikos 2008,117: 1227–1239.

[55] Bascompte J, Jordano P, Melian CJ, Olesen JM. The nested assembly of plant-animal mutualistic networks. Proc Natnl Acad Sci USA 2003,100: 9383–9387. 12881488 10.1073/pnas.1633576100 PMC170927

[56] Klopfer PH, MacArthur RH. On the causes of tropical species diversity: niche overlap. Amer Nat 1961,95: 223–226.

[57] MacArthur RH, Wilson EO. The Theory of Island Biogeography. Princeton: Princeton University Press 1967

[58] Connell JH. Some mechanisms producing structure in natural communities. A model and evidence from field experiments In: Cody ML, Diamond JMCP, editors. Ecology and Evolution of Communities. Harvard University Press pp. 460–490. 1975

[59] Tilman D, Lehman CL, Bristow CE. Diversity-stability relationships: statistical inevitability or ecological consequence? Amer Nat 1998,151: 277–282. 10.1086/286118 18811358

[60] Keddy PA. Assembly and Response Rules—2 Goals for Predictive Community Ecology. J Veg Sci 1992,3: 157–164.

[61] Azeria ET, Kolasa J. Nestedness, niche metrics and temporal dynamics of a metacommunity in a dynamic natural model system. Oikos 2008,117: 1006–1019.

[62] Diamond JM. Assembly of Species Communities In: Cody ML, Diamond JMCP, editors. Ecology and Evolution in Communities. Harvard University Press pp. 342–444. 1975

[63] Gotelli NJ. Ecology—Ecological assembly rules—Perspectives, advances, retreats. Science 1999,286: 1684–1685.

[64] Gotelli NJ, Mccabe DJ. Species co-occurrence: A meta-analysis of J. M. Diamond's assembly rules model. Ecology 2002,83: 2091–2096.

[65] Swenson NG. The Role of Evolutionary Processes in Producing Biodiversity Patterns, and the Interrelationships Between Taxonomic, Functional and Phylogenetic Biodiversity. Amer J Bot 2011,98: 472–480. 10.3732/ajb.1000289 21613140

[66] Mouillot D. Niche-assembly vs. dispersal-assembly rules in coastal fish metacommunities: implications for management of biodiversity in brackish lagoons. J Appl Ecol 2007,44: 760–767.

[67] Law R, Blackford JC. Self-assembling food webs: a global viewpoint of coexistence of species in Lotka-Volterra communities. Ecology 1992,73: 567–578.

[68] Gleason HA. The individualist concept of plant association. Am Midl Natur 1939,21: 92–110.

[69] Shurin JB, Amarasekare P, Chase JM, Holt RD, Hoopes MF, Leibold MA. Alternative stable states and regional community structure. J Theor Biol 2004,227: 359–368. 15019503 10.1016/j.jtbi.2003.11.013

[70] Kadoya T, Suda SI, Tsubaki Y, Washitani I. The sensitivity of dragonflies to landscape structure differs between life-history groups. Landsc Ecol 2008,23: 149–158. 10.1016/j.tree.2007.11.005 18289716

[71] Baselga A. Partitioning the turnover and nestedness components of beta diversity. Global Ecol Biogeogr 2010,19: 134–143.

[72] Tracy CR, Tracy BJ, Dobkin DS. The role of posturing in behavioral thermoregulation by Black Dragons (*Hagenius brevistylus* Selys; Odonata). Physiol Zool 1979,52: 565–571.

[73] Damm S, Dijkstra KDB, Hadrys H. Red drifters and dark residents: The phylogeny and ecology of a Plio-Pleistocene dragonfly radiation reflects Africa's changing environment (Odonata, Libellulidae, *Trithemis*). Mol Phylog Evol 2010,54: 870–882. 10.1016/j.ympev.2009.12.006 20004729

[74] Ferreira-Peruquetti P, De Marco P Jr. Efeito da alteração ambiental sobre comunidades de Odonata em riachos de Mata Atlântica de Minas Gerais, Brasil. Revta bras Zool 2002,19: 317–327.

[75] Remsburg AJ, Olson AC, Samways MJ. Shade alone reduces adult dragonfly (Odonata: Libellulidae) abundance. J Ins Behav 2008,21: 460–468.

[76] Clausnitzer V. Dragonfly communities in coastal habitats of Kenya: indication of biotope quality and the need of conservation measures. Biodiver Conserv 2003,12: 333–356.

[77] Crowley PH, Johnson DM. Habitat and seasonality as niche axes in an odonate community. Ecology 1982,63: 1064–1077.

[78] Covich AP. Geographical and historical comparisons of neotropical stream: biotic diversity and detrital processing in highly variable habitats. J N Amer Benth Soc 1988,7: 361–386.

[79] Melo AS, Froehlich CG. Macroinivertebrates in neotropical streams: richness patterns along a catchment and assemblage structure between 2 seazons. J N Amer Benth Soc 2001,20: 1–16.

[80] Naiman RJ, Mellilo JM, Lock MA, Ford TE, Reice SR. Longitudinal Patterns of Ecossystem Processes and Community Structure in a Subarctic River Continuum. Ecology 1987,5: 1139–1156. 10.1007/BF01020544 24302138

[81] Statzner B, Higler B. Questions and Comments on the River Continuum Concept. Can J Fish Aquat Sci 1985,42: 1038–1044.

[82] Bojsen BH, Jacobsen D. Effects of deforestation on macroinvertebrate diversity and assemblage structure in Ecuadorian Amazonian Streams. Arch Hydrobiol 2003,158: 317–342.

[83] Becker CG, Fonseca CR, Haddad CFB, Batista RF, Prado PI. Habitat split and the global decline of amphibians. Science 2007,318: 1775–1777. 18079402 10.1126/science.1149374

[84] Becker CG, Fonseca CR, Haddad CFB, Prado PI. Habitat Split as a Cause of Local Population Declines of Amphibians with Aquatic Larvae. Conserv Biol 2010,24: 287–294. 10.1111/j.1523-1739.2009.01324.x 19758391

[85] Morton DC, DeFries RS, Shimabukuro YE, Anderson LO, Arai E, Espirito-Santo FB, et al Cropland expansion changes deforestation dynamics in the southern Brazilian Amazon. Proc Natnl Acad Sci USA 2006,103: 14637–14641. 16973742 10.1073/pnas.0606377103 PMC1600012

[86] Biggs TW, Dunne T, Roberts DA, Matricardi E. The rate and extent of deforestation in watersheds of the southwestern Amazon Basin. Ecol Applic 2008,18: 31–48. 10.1890/06-1689.1 18372554

[87] Agarwal DK, Silander JA, Gelfand AE, Dewar RE, Mickelson JG. Tropical deforestation in Madagascar: analysis using hierarchical spatially explicit, Bayesian regression models. Ecolog Model 2005,185: 105–131.

[88] Carvalho FMV, De Marco P, Ferreira LG. The Cerrado into-pieces: Habitat fragmentation as a function of landscape use in the savannas of central Brazil. Biol Conserv 2009,142: 1392–1403.

[89] Bartholomew GA. A matter of size: an examination of endothermy in insects and terrestrial vertebrates In: Heinrich B, editors. Insect Thermoregulation. New York: John Wiley & Sons pp. 46–78. 1981.

